# Retraction: 8-bromo-7-methoxychrysin targets NF-κB and FoxM1 to inhibit lung cancer stem cells induced by pro-inflammatory factors

**DOI:** 10.7150/jca.80863

**Published:** 2022-12-30

**Authors:** Qing Yuan, Min Wen, Chang Xu, A Chen, Ye-Bei Qiu, Jian-Guo Cao, Jian-Song Zhang, Zhen-Wei Song

**Affiliations:** 1 Department of preclinical medicine, Medical College, Hunan Normal University, Changsha, 410013, China; 2 Department of Pharmaceutical Science, Medical College, Hunan Normal University, Changsha, 410013, China; 3 Key Laboratory of Study and Discover of Small Targeted Molecules of Hunan Province, Changsha 410013, China

We feel regret to retract our article (DOI: 10.7150/jca.30143). We cannot revalidate the original images of western blot since most authors have left the lab, being either graduated or re-located. Additionally, we do not have the financial resources to repeat the experiments so that we can submit an erratum. All authors agree to the retraction of this article.

